# Healthcare costs of diabetic foot disease in Italy: estimates for event and state costs

**DOI:** 10.1007/s10198-022-01462-w

**Published:** 2022-05-05

**Authors:** Chiara Seghieri, Francesca Ferrè, Elisa Foresi, Alice Borghini

**Affiliations:** Department EMbeDS, Institute of Management, Scuola Superiore Sant’Anna, Pisa, Italy

**Keywords:** Diabetes, Foot disease, Direct care costs, Population-based study, Tuscany, I1, I18

## Abstract

**Objective:**

This study aimed to estimate healthcare costs of diabetic foot disease (DFD) in a large population-based cohort of people with type-2 diabetes (T2D) in the Tuscany region (Italy).

**Data sources/study setting:**

Administrative healthcare data of Tuscany region, with 2018 as the base year.

**Study design:**

Retrospective study assessing a longitudinal cohort of patients with T2D.

**Data collection/extraction methods:**

Using administrative healthcare data, DFD were identified using the International Classification of Diseases, Ninth Revision, Clinical Modification codes.

**Methods:**

We examined the annual healthcare costs of these clinical problems in patients with T2D between 2015 and 2018; moreover, we used a generalized linear model to estimate the total healthcare costs.

**Principal findings:**

Between 2015 and 2018, patients with T2D experiencing DFD showed significantly higher average direct costs than patients with T2D without DFD (*p* < 0.0001). Among patients with T2D experiencing DFD, those who experienced complications either in 2015–2017 and in 2018 incurred the highest incremental costs (incremental cost of € 16,702) followed by those with complications in 2018 only (incremental cost of € 9,536) and from 2015 to 2017 (incremental cost of € 800).

**Conclusions:**

DFD significantly increase healthcare utilization and costs among patients with TD2. Healthcare costs of DFD among patients with T2D are associated with the timing and frequency of DFD. These findings should increase awareness among policymakers regarding resource reallocation toward preventive strategies among patients with T2D.

**Supplementary Information:**

The online version contains supplementary material available at 10.1007/s10198-022-01462-w.

## Introduction

Worldwide, diabetes is a major health public health concern, with > 460 million adults living with diabetes, which is estimated to increase to 700 million in 2045. Moreover, in 2019, the healthcare expenditure costs for diabetes were ≥ USD 760 billion [1].

Some of these expenditures result from complications, including cardiovascular events, kidney failure, and foot diseases (i.e., infections, ulcers, and gangrene). Diabetic foot disease (DFD) is among the most severe and costly long-term diabetic complications. The most significant manifestation of diabetic foot problems, such as amputations significantly contribute to both mortality and morbidity among patients with diabetes and exert a considerable financial burden on patients and healthcare systems. Recent findings based on Global Burden of Disease 2016 data indicated that approximately 131.0 million (1.77%) people worldwide presented DFD in 2016, including 105.6, 18.6, and 6.8 million individuals with neuropathy only, foot ulcers, and amputations, respectively [2]. These complications have led to approximately 16.8 million years lived with disability (YLDs), which corresponds to 59% of diabetic YLDs. Among DF problems, foot amputation has a higher impact on outcomes, with more than half of the individuals with a major amputation being likely to die within 5 years and their survival rate being lower than the 5-year survival rate of patients with all cancers [3].

In 2019, diabetes-related expenditure in Europe was approximately USD 161 billion, which accounts for 21% of the global diabetes-related expenditure. A recent review of studies assessing the costs for DFD which includes amputation, ulcers, gangrene, across European countries reported increasing costs over time, especially for amputation, which widely varied across countries [4]. Petrakis and colleagues also reported in a worldwide systematic review of cost studies in DFD the substantial costs and further healthcare burden for people with diabetes due to disease complications [5]. As expected, they found that amputations due to suboptimally treated foot infections contribute to the already high rates of hospitalizations and readmissions. The cost of amputation ranges between $35,000 and $45,000 in the developed countries.

DFD results in greater expenditure than major diseases, including cancer, lung disease, and depression [3, 6]. In 2014, a comparison of the direct costs of various conditions in patients with diabetes in the UK revealed that from 1997 to 2007, the annual average amputation costs (£ 12,245) were much higher than those for non-fatal ischemic heart disease (£ 10,631), non-fatal stroke (£ 7,824), non-fatal myocardial infarction (£ 8,342), and heart failure (£ 4,170) [7]. Moreover, these patterns have been reported in other German studies [8].

Strategies, including prevention, patient and healthcare staff education, multidisciplinary treatment, and regular close monitoring, can significantly prevent and reduce DFD, especially amputations [9, 10].

In 2019, Italy, which has a regionally based National Health Service (NHS) with universal coverage that is largely free of charge at the delivery point, was among the top five European countries in terms of the number of people with diabetes (20–79 years) (about 3.7 million people with diabetes) (IDF 2019) [1]. Although there is a national strategy for improving the management of patients with diabetes (see the 2016 National Plan for Chronic Diseases), there remains unwarranted geographical variations in the management of diabetes and related complications, as well as in the costs of DFD across and within Italian regions [11, 12].

Despite the significance of the increasing prevalence and costs of diabetes and its complications, there remain scarce population-based studies on the economic impact of diabetes and, in particular, DFD; moreover, they are often limited to country-level estimates or prospective data collection from selected diabetic centers (e.g., the Eurodiale Study) [4, 13, 14].

Given these premises, we analyzed the annual costs of DFD in a large population-based cohort of people with type-2 diabetes (T2D) living in the Tuscany region (Italy). Specifically, this work aims at estimates healthcare costs for the management of DFD in patients with T2D considering two cost components: state and event costs. Event costs represent the complication costs associated with resources use in the base year that is specific to the acute episode (managed both in an inpatient or outpatient setting) and any subsequent care occurring in the same year. State costs refers to the management costs for years subsequent to the event year and reflect the typical utilization of health care services for the continuous management of complications experienced in the years before.

## Materials and methods

Studies on cost and service utilization for DFD have predominantly estimated direct medical costs in continental Europe, Northern America or Australia using heterogeneous approaches often limited to cost-effectiveness studies comparing different treatment options [5]. We conducted a population-based study using individual-level administrative data from the Tuscany region (Italy) analysing costs in the year 2018.

Italy’s health-care system is a regionally based National Health Service, which provides universal coverage largely free of charge at the point of delivery [15]. Tuscany is a large region in central Italy characterized by a non-competitive health system, where patients are free to choose any provider. The regional healthcare system comprises three local health authorities, four teaching hospitals and 26 health districts, which oversee the organization and delivery of services for local health networks, social care and social integration. Tuscany has a total population of over 3.7 million inhabitants (about 6.2% of the Italian population) (Demo Istat). Tuscany, mainly public providers deliver hospital and outpatient/walk-in care (over 90% of all outpatient and laboratory services were provided either by public local health authorities or by public hospitals). Inpatient care is reimbursed using Diagnosis Related Groups (DRGs), while outpatient care is reimbursed using a tariff per unit of care (prospective payment system) and patients are asked to contribute to the cost via co-payments with exemptions based on gross family income, age, chronic and rare conditions and disabilities. Certified diabetic patients are entitled to co-payment exemptions. Also, for pharmaceutical expenditures related to drugs for the treatment of chronic conditions patients are exempted to co-payments.

The Tuscany healthcare administrative databases contain information regarding all public and private accredited healthcare providers. The individual-level databases used in this study included (i) hospital inpatient data; (ii) emergency care data; (iii) outpatient care data; (iv) drug prescription data; (v) exemption data; and (vi) a registered person database containing socio-demographic information on all residents enrolled in the Tuscan healthcare system, including sex, date of birth, and date of death. Different administrative databases were linked at the individual (patient) level using a unique identifier. The data were anonymized at the Regional Health Information System Office, where each patient was assigned a unique identifier to prevent tracing of the patient’s identity and other sensitive data. This study was conducted in compliance with Italian law on privacy; approval by the conjoint ethics committee of Scuola Superiore Sant’Anna di Pisa and Scuola Normale di Pisa (Italy) was also obtained (Delibera no. 20/2020).

To estimate the DFD-related costs in the year 2018, we considered all residents living in Tuscany, Italy who entered the T2D cohort from 2010 to 2014, and alive on December 31st, 2018. T2D account for 95% of all patients with diabetes, in the Tuscany region.

These selection criteria ensured that the patients’ complications during 3 years before the base year (2018) were related to diabetes (i.e., did not occur before diabetes onset) [16]. This T2D cohort was identified by applying a disease-specific algorithm [17, 18] to health administrative data obtained between 2010 and 2018. Specifically, individuals who met the following criteria were considered as patients with diabetes: age > 35 years and patients with an exemption for diabetes within the previous 10 years or a history of hospitalization for diabetes within the last 5 years or anti-hyperglycemic medication prescriptions within 1 year or having received outpatient services for diabetes within 1 year.

Based on a previously described methodology [19], we identified the presence of DFD using the International Classification of Diseases, Ninth Revision, Clinical Modification (ICD-9-CM) codes, as well as the procedure code. Specifically, a patient with T2D was considered as having experienced DFD between 2015 and 2018 if they had been hospitalized for at least one of the following diagnoses or procedure [19]: foot ulcers (ICD9-CM codes: 440.23, 707.14–5), Charcot neuroarthropathy (ICD9-CM codes: 713.0, 713.5, 713.8), procedures regarding major and/or minor lower extremity amputations (ICD9-CM codes: 84.10–84.19), revascularizations (ICD9-CM codes 39.25, 39.29, 39.50, 39.90), gangrene (ICD9-CM codes: 785.4, 040.0, 440.24), and foot infections (ICD9-CM codes: 681.10, 681.11, 681.9, 682.6, 682.7, 682.9, 730.07, 730.17, 730.27). In addition, we also compared DFD costs with costs of other diabetes-related complications including cardiovascular events, eye condition due to high blood sugar from diabetes and chronic kidney disease (CKD) (ICD9-CM codes for the identification of these complications are reported in Appendix 1).

### Variables and statistical approach

We calculated the direct care costs in the year 2018 for each patient with T2D by considering cost information obtained from the following administrative databases: inpatient care (DRG tariffs), drugs (net costs), and outpatient and emergency services (outpatient tariffs). All costs are expressed in 2018 euros. Moreover, we considered demographic variables (age and sex) from regional databases. First, we analysed the different healthcare costs for 2018 of T2D patients considering number and type of complications. Specifically, we defined five patients’ groups ranging from patients with no complication (group 1) to patients with two or more complication distinguishing between DFD complications and other diabetes-related complications (group 5). Subsequently, we focused on estimation of DFD costs. Given the low number of zero costs (2.5%), care costs for DFD in the T2D population were estimated using a GLM. To determine the correct model specification, we employed the modified Park test [20] and Pregibon’s Link test [21] which demonstrated that the Poisson-Log link model was adequate compared with the other distribution types. Moreover, regarding healthcare cost estimation, we considered 2018 as the base year and divided the costs of DFD into two components: (i) event costs, which was defined as the complication costs accrued in 2018 when the patient first experienced DFD; (ii) state costs, which was defined as the costs accrued in 2018 related to the management of DFD problems experienced from 2015, 2016, or 2017 [16]. Therefore, we modelled the different impact of timing on costs by including a variable in the GLM model that indicated whether patients belong to any of the following groups: T2D population without DFD from 2015 to 2018 (reference group), T2D population with DFD in 2018 but not from 2015 to 2017 (i.e., those who incurred the event cost of the complication in 2018), T2D population with DFD at some point between 2015 and 2017 (i.e., those who incurred the state cost of the complication in 2018), and T2D population who experienced DFD in 2015–2017 and in 2018. In addition, this model was adjusted for age and sex. We decided not to adjust for comorbidities to avoid over-adjustment [22].

Finally, we calculated the incremental costs for foot complications by subtracting one from the GLM coefficient expressed as incidence rate ratios and multiplying them by the estimated mean annual healthcare costs of a 70-year-old man without foot complications.

In all analyses, statistical significance was set at a *p* value of < 0.05. Moreover, confidence intervals were calculated at 95%. All statistical analyses were performed using SAS for Windows Ver. 9.3 (SAS Institute, Cary, NC, USA) and STATA Software v14.

## Results

A total of 51,748 patients with T2D resided in Tuscany on December 31, 2018. Among them, 53% were male and the majority (88.46%) did not experience diabetes-related complications from 2015 to 2018; moreover, the average age was 69 years. The results also showed that men were more likely than women to have diabetes-related complications (13.16% vs 9.69%; *p* < 0.001). In addition, compared with patients without a diabetes-related complication, patients with a complication were older (75.18 ± 10.97 vs 68.26 ± 12.16; *p* < 0.001). The mean annual total healthcare costs for men were slightly higher than for women (€2590 vs €2222; *p* < 0.001) and increasing by age group (see Appendix 2). Table 1 reports healthcare costs incurred in the year 2018 comparing patients with and without diabetes-related complications differentiating by number and type of complication. Specifically, the following five groups of T2D patients were analysed: (a) patients who had no history of diabetes-related complications from 2015 to 2018 (group 1); (b) patients with one of the selected diabetes-related complication from 2015 to 2018 excluding DF complications (group 2); (c) patients who had history of DFD from 2015 to 2018 and no other complication (group 3); (d) patients who had history of two or more diabetes-related complications from 2015 to 2018 excluding DFD (group 4); patients with two or more complication including DFD from 2015 to 2018 (group 5). Among the patients with complications (*N* = 5,972), 730 (12.22%) patients experienced at least one DFD in 2015–2018 (group 3 and 5, Table 1). Out of these 730 patients, 474 experienced at least one DFD combined with other complications (group 5) during 4 years considered in the analysis, revascularization, and foot ulcers (37%) being the most recurrent combination. The remaining 5,242 patients (87.77%), in the same period, had any of the 5 diabetes-related complications excluding the foot related ones.

**Table 1:** Total annual healthcare costs and percentage of costs among type of healthcare services for T2D patients without complications (Group 1) and with complications (Group 2 to 5)

	Group 1	Group 2	Group 3	Group 4	Group 5
Number of patients	45,776	4,135	256	1,107	474
Number of complication	None	1	1	> 1	> 1
DFD included	n.a	No	Yes	No	Yes
Total annual healthcare costs (million)	€85.4	€24.4	€1.2	€9.1	€5.0
Hospitalization costs (%)	35%	61%	61%	64%	59%
Emergency department service costs (%)	2%	1%	1%	2%	1%
Outpatient visit and diagnostics costs (%)	17%	14%	11%	15%	27%
Drugs costs (%)	46%	24%	27%	19%	13%

Costs for patients with complications are analysed by number and type of complications

*DFD* Diabetic Foot Disease

With regard to 2018 medical costs incurred by the different groups of T2D patients shows that drug therapies were the major cost drivers for T2D patients not showing complications in the period of observation (group 1), while T2D suffering any complication resulted in significantly higher hospitalization costs (groups 2–5). Inpatient costs for managing diabetic complications were significantly higher compared to outpatient expenses, thus representing the key driver of complication costs. When comparing patients by number of complications the cost composition is very similar between T2D patients suffering one complication (group 2 and 3) and patients suffering more than one complication (group 4 and 5), so number of complications do not seem to impact healthcare cost composition. However, it is interesting to note that T2D patients with more than one complication including DFD (group 5) registered a higher share of outpatient costs compared to the other groups of patients with complication often due to expenses attributed to the management of events in ambulatory settings, such as foot ulcers and revascularisations. Cost for emergency care is very limited in all patient groups (< 3%).

**Fig. 1:**
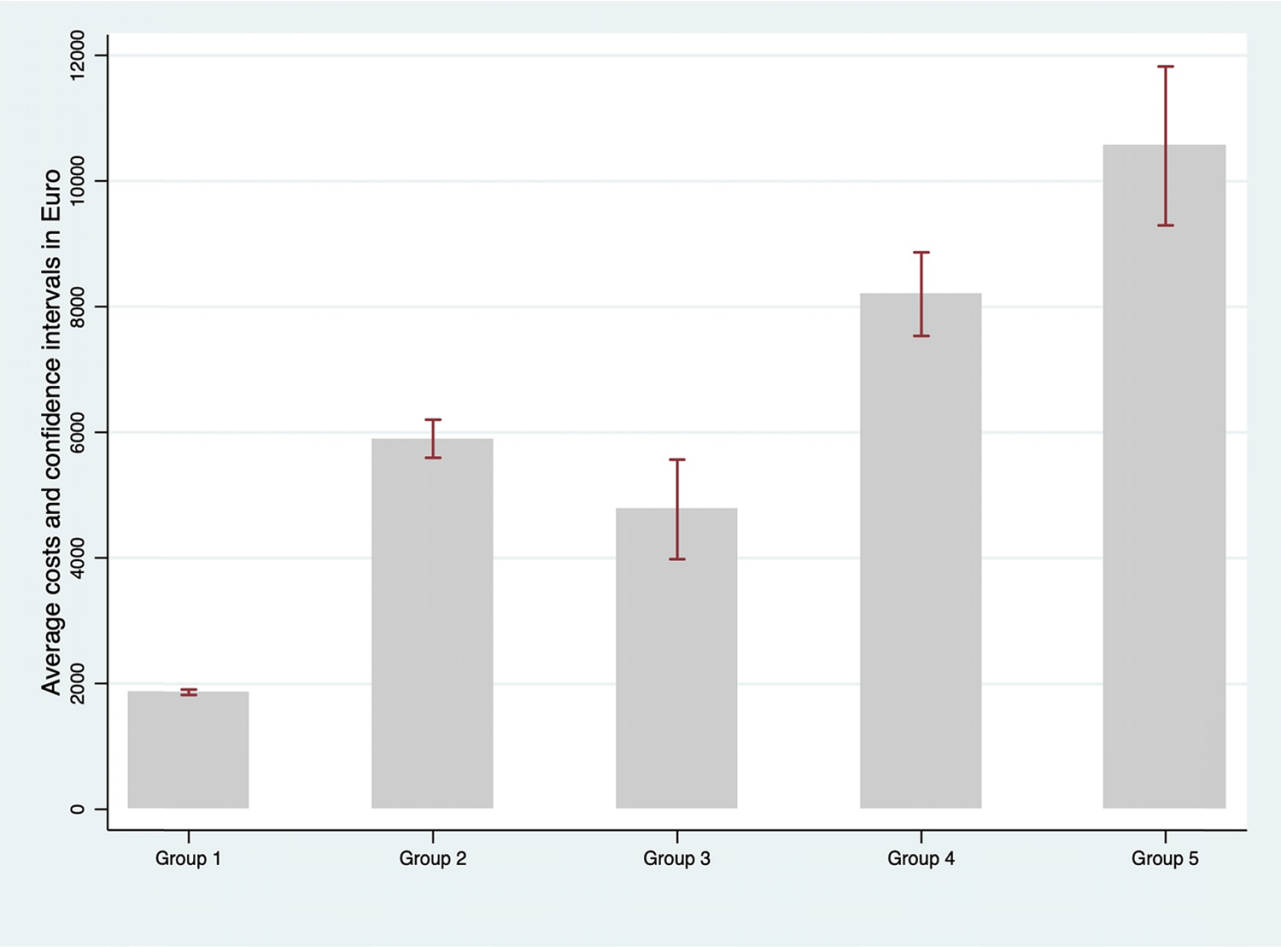
Per-capita average healthcare costs (Euro) for 2018 for the five patient groups and 95% confidence intervals

The total expenses for acute, emergency, outpatient, and drugs for T2D patients in Tuscany resulted in 125.1 million euros in 2018. The annual per-capita direct health care costs for the five groups of patients are summarized in Fig. 1. Per capita annual costs for the care of patients without complication (group 1) are significantly less than patient suffering any diabetes-related complications (€ 1867, CI: [€1824, €1911 vs €6648, CI: [ €6380, €6916]). Healthcare costs for the management of only one diabetes-related complications (comparing DFD vs other complications) are similar in cost composition (Table 1) but is higher in absolute value the costs for managing other diabetes complication compared to foot-related ones (€ 5900, CI [ €5597, €6202] vs €4774, CI:[ €3982, €5567]). Total mean costs for patients with more than one complication substantially increase above € 8,000 per year. Specifically, when comparing the medical cost of patients in group 4 and group 5 the presence of DFD significantly increase the direct healthcare costs (€ 8201, CI [€7536, €8867] vs €10,561, CI [€9297, €11,826]). These numbers shade some lights on the contribution of DFD on overall costs for T2D complications.

A specific analysis of the 730 patients with DFD revealed that 114 patients had an amputation (15,6%) in the period of analysis (2015–2018) and the respective annual per-capita costs was calculated at € 12,146 compared to an annual patient cost of € 7,864 for the remaining 616 patients incurred in other DFD excluding amputation. This evidence confirms amputations to be the major costs driver of DF problems.

When considering the costs associated to DFD only, the GLM analysis results (Table 2), show that males and the elderly had significantly higher costs than their counterparts. Patients who experienced at least one foot-related complication in 2015–2018 had significantly higher costs in 2018 than those who did not have any DFD complication in 2018 or during the years 2015–2017 (reference group).

**Table 2:** Results of the GLM model

	Coefficients^a^
Group (ref = no DFD)	
Event	4.68*** (0.31)
State	1.31*** (0.12)
Event and state	7.45*** (0.53)
Male	1.18*** (0.03)
Age classes (ref ≤ 55)	
55–64	1.12*** (0.06)
65–74	1.42*** (0.07)
75–84	1.68*** (0.08)
85 +	1.54*** (0.08)
Intercept	1545.76*** (68.39)
* N*	51,748

*Shows the coefficients in terms of incident rate ratios and cluster-robust standard errors

*Statistical significance at the 10% level

**Statistical significance at the 5% level

***Statistical significance at the 1% level

**Fig. 2:**
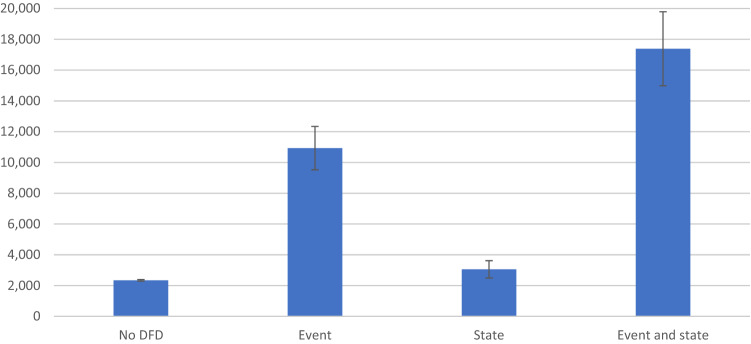
Estimated annual costs (Euro) and 95% CI in 2018 for patients with TD2 without and with foot complications (event, state, both state and event) between 2015 and 2018. *Event costs* represent the complication costs accrued in the base year when the patient first experienced that complication. *State costs* refers to the costs accrued in the base year related to the management of complications experienced in the years before

Specifically, using the mean value for estimation, the annual costs in year 2018 ranged from € 2,334 (95% CI 2.284–2.383) in patients with T2D without DFD to € 10,931 (95% CI 9.525–12.336) for patients with DFD in 2018 who had no history of that complication from 2015 to 2017 (i.e., those who incurred the event cost of the complication in 2018), € 3,055 (95% CI 2.493–3.617) for those with the presence of the DFD at some point between 2015 and 2017 (i.e., those who incurred the state cost of the complication in 2018) and € 17,390 (95% CI 14.987–19.794) for those who incurred in DFD both between 2015 and 2017 and in 2018 (Fig. 2).

Similar results were obtained when considering incremental costs, patients who experienced DFD both in 2015–2017 and in 2018 incurred the highest incremental costs (€ 16,702), followed by those who experienced DFD in 2018 only (incremental cost of € 9,536) and 2015–2017 (incremental cost of € 800).

## Conclusions and discussion

The cost related to complications is the most significant contributor to the costs related to diabetes requiring expensive medical interventions and treatment [23, 24]. This study provides a detailed estimation of the direct costs for T2D and diabetes-related complications considering both DFD and other major complications in an Italian region in 2018 considering state and event costs. The Tuscany Region has about 4 million inhabitants; moreover, in 2018, there were 730 (1.41%) patients with T2D and DFD, which corresponded to approximately € 6.2 million direct costs (5% of the total costs of care of patients with T2D). We used the ICD-9-CM codes to identify the occurrence of DFD of interest (foot ulcers, Charcot neuroarthropathy, major and/or minor lower extremity amputations, revascularizations, gangrene, and foot infections) and other major complications including cardiovascular events, CDK and retinopathy. Through retrospective database analysis using a longitudinal cohort of patients with T2D with 2018 as the base year, we found that the average direct cost for patients with one diabetes-related complication was almost 3.5 times that of patients without any complications. Moreover, a majority of the costs resulted from hospitalizations (about 60%) for patients with complications and DFD accounting for few events but significantly contributing to total annual expenses (group 3 and 5). Moreover, amputations due to suboptimally treated foot infections contribute to the already high rates of hospitalizations and readmissions confirming evidence from the international literature [5].

In 2018, the adjusted event cost of DFD (cost related to first experienced DFD) was 4.6 times that of patients without DFD and 3.7 times the adjusted state costs in 2018 (costs accrued in 2018 associated with managing DFD experienced by the patient since 2015, 2016, or 2017). On the other hand, consistent with the findings by Kähm et al. (2018), DFD were likely to affect the total direct health care costs even years after the event, with the state costs in 2018 being 1.31 times higher than those of patients without DFD.

Moreover, there were differences among women and men in different age groups in the complication costs; specifically, males and the elderly had significantly higher costs compared with other categories.

These findings are consistent with previous reports. In the UK, the direct costs of diabetes in 2010/11 were £9.8 billion, with approximately 80% being spent on complications (https://www.diabetes.co.uk/cost-of-diabetes.html—report on costs). A German study using claims data reported that T2D complications significantly affected the total direct health care costs even years after the event, with variations according to demographics and complication type [22]. This is consistent with our findings, which highlights the incremental cost among the three groups experiencing complications. Our findings indicate that the costs of DFD among patients with T2D are associated with the timing and frequency of experiencing DFD. Patients who developed DFD in 2015–2017 showed the lowest incremental cost, followed by those who experienced complications in 2018 only; contrastingly, those who experienced DFD in 2015–2017 and 2018 incurred the highest incremental costs compared to those who did not experience any DFD problem in 2015–2018.

The diabetic population has a significant economic burden, especially with respect to foot-related complications, even compared with other major diseases, which indicates a deficiency of cost-preventive strategies to treat patients [3]. Several risk factors, including overweight, obesity, nutrition, and physical inactivity, can be modified through effective preventive strategies and lifestyle changes. Effective management of the increasing T2D population is a priority in numerous countries [25], which usually involves a considerable self-care amount. Previous studies have indicated that improvements in the control and primary care of diabetes could reduce the direct costs for managing complications, as well as the decrease of indirect costs (e.g., production losses due to work absence and impairment, early retirement) [23].

Indeed, according to the most recent guideline an effective preventive strategy has to be personalised and tailored to patients’ needs and their clinical conditions [10]. Considering DFD, patients will benefit most from the preventive strategies which aim at preventing foot ulcers, e.g., identifying the at-risk foot, regularly inspecting and examining the at-risk foot or educating patient and family; these interventions impact less in term of costs and are related to better quality of life.

However, for those patients who experienced any DFD complications for the first time or have complications from several years, it should be considered both clinical treatment of complication and preventive interventions to avoid the occurrence of new complications [10]. As demonstrated in the result sections, the costs related to patients with complications experienced both in the period 2015–2017 and in 2018 (event and state cost) are much higher compared to the one without complications (Fig. 2).

Therefore, considering the great and increasing number of TD2 patients, efforts are needed to disseminate and implement the most recent and evidence based preventive strategies as well as treatment programs, including self-care, among patients with T2D that may have the potential to avoid or halt DFD problems (e.g., ulcer) [10]. At system level, increased integration among settings of care (e.g., preventive and acute care), professionals (e.g., primary care physicians and specialists) and disciplines working on diabetes care (e.g., consultants in diabetology and vascular surgery) is of priority [12]. These interventions will contribute to reduce the cost related to treatment, as well as the risk of associated disabilities and severe outcomes; and, in parallel, improve the quality of life in the T2D population [26–28].

This study had some limitations. First, we only used administrative data sources. Although they are widely available at a reasonable cost, only allowed identification and estimation of direct medical costs and, therefore, underrepresents the economic care burden of DFD. Moreover, administrative data lack information about the clinical aspects of diabetic patients, such as severity and duration of the disease (there is no information about the date of onset of the disease) thus limiting the comparability of individuals by stage of disease. Second, the complications were identified based on the ICD-9-CM codes listed in administrative data, which might have had errors and incompleteness, resulting in the misclassification of patients in each complication group. However, the complications were identified as previously reported [19].

Regarding the strengths, this study covered a homogeneous, large, and well-characterized population with diabetes, with a long-term observation period.

Our findings highlight the importance of prevention in terms of value for patients with T2D, society, and the healthcare system, regardless of the health system model. Indeed, implementing preventive strategies to provide optimal care to patients with T2D upon initial diagnosis could reduce the onset and risk of complications. Specifically, the results of this study indicate the need for more efforts toward improving secondary prevention strategies in patients with diabetes to prevent avoidable and costly complications, especially regarding DFD. Shifting money from treating complications to secondary prevention, including implementing more well-equipped outpatient ambulatories, increasing advice and education, and personalized care pathways for patients at a high complication risk, could provide high-value interventions for patients with diabetes, as well as facilitate the sustainability of healthcare systems. This is especially true for amputations, which disproportionally contributes to diabetes-related costs and reduces patients’ quality of life.

To promote sustainability and better use of resources, several approaches should be considered, including evidence-based clinical pathways for diabetes care, encouraging healthcare workers working in different setting of care (e.g., primary care, hospital, public health), to be responsible and accountable for patients with diabetes they have in common [29] also with new governance and reimbursement models [30], engaging patients in shared decision-making, and considering patients’ goals and preferences [31, 32]. In general, there is a need to improve the treatment value in the whole population, which requires adopting a population medicine approach [33]. To foster a population-based approach improvements are expected also in planning and control systems [34], where target setting and monitoring activities should use population outcome performance information (e.g., reducing avoidable hospitalization), which better represent the value creation process [35]. Indeed, these metrics reflect preventive activities that have the greatest potential for yielding population benefit.

In conclusion, the complications of diabetes present a substantial economic burden especially for those related to DFD which significantly contributes to an increased use of healthcare services both hospital and outpatient and associated costs. In addition, healthcare costs of DFD among patients with T2D appear linked with the timing and frequency of DFD. The first experience of a foot complication (event) is per se expensive and higher compared to state costs showing the importance of increasing efforts in avoiding the first occurrence of complications. For many complications the resulting management costs persist after the event, because it has placed the patient in a new health status, and, if not correctly managed, might increase the likelihood of new event. Therefore, significant higher costs (state and event costs) will arise. Efforts to avoid or reduce complications would be beneficial not only to the patient, but also to health care systems. Indeed, findings should increase awareness among policymakers regarding resource reallocation toward comprehensive approaches targeting prevention, primary care and screening, and patient education in the management of their disease.

## Supplementary Information

Below is the link to the electronic supplementary material.

Supplementary file1 (DOCX 12 kb)

Supplementary file2 (DOCX 14 kb)
