# Supplementary material for: Healthcare costs of diabetic foot disease in Italy: estimates for event and state costs
Source: Eur J Health Econ. 2022 May 5;24(2):169–77. doi: 10.1007/s10198-022-01462-w (PMC9985574; doi:10.1007/s10198-022-01462-w)
Supplement: Supplementary file 1 — Supplementary file1 (DOCX 12 kb) [file 10198_2022_1462_MOESM1_ESM.docx]

| Ischemic stroke | 430.xx; 431.xx; 432.xx; 434.xx; 436.xx |
| --- | --- |
| Myocardial infarction | 410.xx |
| Chronic heart failure (CHF) | 401.91; 402.01; 402.11; 402.91; 404.01; 404.03; 404.13; 404.93; 428.0; 428.1; 428.9 |
| Retinopathy | 362.01; 362.02; 362.03; 362.04; 362.05; 362.06; 362.81; 362.82; 362.83; 362.84 |
| Chronic Kidney Disease (CKD) | 585.x |

**Appendix 1**. ICD9-CM codes for the identification of other diabetes-related complications
