# Supplementary material for: Healthcare costs of diabetic foot disease in Italy: estimates for event and state costs
Source: Eur J Health Econ. 2022 May 5;24(2):169–77. doi: 10.1007/s10198-022-01462-w (PMC9985574; doi:10.1007/s10198-022-01462-w)
Supplement: Supplementary file 2 — Supplementary file2 (DOCX 14 kb) [file 10198_2022_1462_MOESM2_ESM.docx]

|  | N | % patient with at least 1 diabetes-related complication |  | Mean costs (euro) | Std. Dev. Costs (euro) |  |
| --- | --- | --- | --- | --- | --- | --- |
| **Sex:** |  |  | p < 0.001 |  |  | p < 0.001 |
| Male | 27.630 | 13,2 |  | 2590,6 | 6463,8 |  |
| Female | 24.118 | 9,7 |  | 2222,2 | 5226,4 |  |
| **Age classes:** |  |  | p < 0.001 |  |  | p < 0.001 |
| <=55 | 6.631 | 4,0 |  | 1746,0 | 6330,4 |  |
| 55-64 | 11.018 | 6,8 |  | 1966,9 | 5497,4 |  |
| 65-74 | 15.902 | 10,0 |  | 2495,5 | 6293,8 |  |
| 75-84 | 13.054 | 16,1 |  | 2941,6 | 6005,6 |  |
| 85+ | 5.143 | 24,4 |  | 2691,2 | 4547,0 |  |

**Appendix 2.** Distribution of T2D patients and costs by gender, age, and presence of at least one diabetes-related complication
